# Antidiabetic, Lipid Normalizing, and Nephroprotective Actions of the Strawberry: A Potent Supplementary Fruit

**DOI:** 10.3390/ijms18010124

**Published:** 2017-01-11

**Authors:** Pallavi Mandave, Suresh Khadke, Manjiri Karandikar, Vijaya Pandit, Prabhakar Ranjekar, Aniket Kuvalekar, Nitin Mantri

**Affiliations:** 1 Interactive Research School for Health Affairs, Bharati Vidyapeeth University, Katraj, Pune, Maharashtra 411043, India; mandavepallavi@gmail.com (P.M.); spkhadke@gmail.com (S.K.); pranjekar@gmail.com (P.R.); aniket.kuvalekar@bharatividyapeeth.edu (A.K.); 2 Department of Pathology, Bharati Vidyapeeth Medical College, Bharati Vidyapeeth Deemed University, Pune-Satara Road, Pune, Maharashtra 411043, India; manjiri.karandikar@bharatividyapeeth.edu; 3 Department of Pharmacology, Bharati Vidyapeeth Medical College, Bharati Vidyapeeth Deemed University, Pune-Satara Road, Pune, Maharashtra 411043, India; vijaya.pandit@bharatividyapeeth.edu; 4 School of Science, RMIT University, Melbourne 3000, Australia

**Keywords:** β-cell regeneration/protection effects, antihyperglycemic activity, normolipidemic activity, oxidative stress, strawberry

## Abstract

The study was designed to assess the effect of different strawberry extracts on glucose levels, lipid profiles, and oxidative stress in nicotinamide-streptozotocin (NIC-STZ) induced diabetic rats. The associated changes were evaluated through biochemical, molecular, and histological assays. Diabetes was induced by intraperitoneal injection of STZ to albino Wistar rats after treatment with nicotinamide. Aqueous, hydroalcoholic, and alcoholic strawberry extracts were administrated orally to diabetic rats. Treatment of strawberry extracts improved lipid profile, liver function, and serum creatinine and led to a significant increase in antioxidant status in diabetic rats. Real-time PCR expression analysis of genes from the liver of animals treated with strawberry extracts exhibited downregulation of several fatty acid synthesis genes, transcription factors, such as Sterol regulatory Element Binding Transcription factor (*SREBP*) and Nuclear Factor-κβ (*NF-κβ*), and inflammatory markers, like Interleukin 6 (*IL6*) and Tumor Necrosis Factor-α (*TNF-α*). Strawberry extracts also upregulated liver Peroxisome Proliferator Activated Receptor-γ (*PPAR-γ*). Histological examination confirmed the nephroprotective and β-cell regeneration/protection effects of strawberry extracts. The present study demonstrates several beneficial effects of strawberry extracts along with its probable mechanism of action.

## 1. Introduction

Diabetes mellitus (DM) is characterized by hyperglycemia due to disturbance in the metabolism of carbohydrates, fats, and proteins, resulting from defects in insulin secretion, insulin action, or both [1,2]. Currently, there are over 150 million diabetics worldwide and this number is likely to increase with increase in sedentary lifestyle, consumption of an energy-rich diet, and obesity [3].

Current therapeutic strategies for type 2 diabetes are limited and involve insulin and oral antidiabetic agents that stimulate pancreatic insulin secretion, reduce hepatic glucose production, delay digestion and absorption of intestinal carbohydrates, or improve insulin action. These drugs are reported to have several adverse side effects and hence there is a growing interest in anti-hyperglycemic agents from natural products, especially those derived from plants. Plant sources are usually considered to be non-toxic, with fewer side effects than synthetic sources [4]. Therefore numerous plant-based therapeutic agents/strategies are being examined for the treatment of type 2 diabetes.

The importance of medical nutrition therapy (MNT) is recognized as one of the cornerstones ofthe treatment of Type 2 Diabetes melitus (T2DM) [5,6,7]. Several evidence-based nutrition guidelines have been published indicating the impact of diet quality and quantity on T2DM [5,6,8]. A variety of fiber-rich foods like fruits and vegetables are generally recommended in these guidelines [5,8]. Fruits contain a wide variety of specific bioactive substances with multiple activities like antioxidant, anti-inflammation, and improving endothelial function [9,10,11]. High fruit intake has been shown to reduce the risk of cardiovascular diseases [12,13] and some cancer types [14].

The strawberry (*Fragaria x ananassa*, Dutch.) is one of the most commonly consumed berries worldwide [15]. A variety of nutritional and bioactive compounds from strawberry-like flavonoids, anthocyanins, and non-flavonoid condensed tannins (ellagitannins) have recently attracted growing attention for use innutritional medicine [16,17,18]. A few studies have substantiated the antioxidant effects of whole berry fruits or individual anthocyanins [19,20,21] and also their cytoprotective effects via the activation of antioxidant defense [22]. Ample literature is available examining the effects of strawberries or their extracts, mainly on cardiovascular diseases and diabetic complications. Strawberry juice significantly inhibits free radicals [23] and reduces ox-low-density lipoprotein-induced proliferation of rat aortic smooth muscle cells [24]. Ellagic acid, a component from strawberry fruit, reduces oxidative stress and atherosclerotic lesion formation in hyperlipidemic rabbits [25]. Apples and strawberries are also known to be the largest contributors of cellular antioxidant activity among all fruits consumed [26]. Pinto et al. [27] reported in vitro anti-hyperglycemic and anti-hypertensive effects of Brazilian strawberries. They showed the inhibitory activity of strawberry ellagic acid derivatives against α-amylase, α-glucosidase, and angiotensin I-converting enzyme for the potential management of hyperglycemia and hypertension. Freeze-dried strawberry powder has been shown to reduce obesity and improve glycemic control in mice fed a high-fat diet [28]. Anthocyanin extracts from strawberries caused upregulation of anti-inflammatory adiponectin gene in isolated rat adipocytes and in white adipose tissue in mice [29]. A recent report by Abdulazeez [30,31] has even suggested complete reversal of alloxan-induced diabetes and its complications after the administration of a powder from freeze-dried strawberries. None of the above reports have analyzed the effects of interventions at the molecular level. The present report is a comprehensive analysis of the antioxidant, anti-inflammatory, and anti-hyperglycemic effects of different strawberry extracts at the biochemical and molecular levels and their probable mechanism of action. Flavonoids are soluble in water as well as in organic solvents. Flavonoid glycosides are readily soluble in water, methanol, and ethanol, while flavonoids aglycones are only soluble in methanol and ethanol [32,33]. Hence, aqueous, hydroalcoholic, and alcoholic extracts were used in this study.

In our previous study, we characterized in vitro the antioxidant, anti-diabetic, and anti-glycation activity of strawberry extracts [34,35,36]. In vitro anti-diabetic activity was evaluated by the ability of the extract to inhibit α-amylase and α-glucosidase enzymes. The present study builds on previous work by comprehensively evaluating the in vivo anti-diabetic effects of aqueous, hydro-alcoholic, and alcoholic strawberry extracts. The extracts were evaluated against the NIC-STZ induced diabetes in Wistar rats. The effects of extracts were studied at biochemical, histological, and molecular levels. The probable molecular mechanism of the strawberry extract was also evaluated.

## 2. Results

### 2.1. Animal Observations

The food and water intake of animals between different groups was significantly different and is represented in Table S1. NIC-STZ rats showed a significant increase in food (*p* ≤ 0.05) and water intake (*p* ≤ 0.01) but had reduced body weights. Strawberry aqueous and hydro-alcoholic extracts interventions non-significantly decreased feed intake as compared to diabetic rats. The water intake of the strawberry-extract-treated group showed a significant decrease (*p* ≤ 0.05 or *p* ≤ 0.01) as compared to diabetic rats.

### 2.2. Strawberry Extracts Modulate Serum Glucose Levels

Table 1 indicates the serum glucose from different groups in blood samples collected at different time intervals. From the table it can be seen that NIC-STZ rats had significantly (*p* ≤ 0.01) high glucose levels. These high glucose levels were significantly reduced after administration of strawberry extracts for four weeks. Animals treated with strawberry water extract had the lowest blood glucose levels.

### 2.3. Strawberry Extracts Improve Liver Function

Table 2 provides results from liver function tests in control and treatment groups. NIC-STZ rats displayed a significant (*p* ≤ 0.01) increase in their serum serum glutamic oxaloacetic transaminase (SGOT), serum glutamic pyruvic transaminase (SGPT), alkaline phosphatase (ALP), and bilirubin levels. Aqueous and hydro-alcoholic extracts of strawberry significantly lowered (*p* ≤ 0.01) the serum SGOT and SGPT levels as compared to NIC-STZ control rats. Strawberry extracts lowered (*p* ≤ 0.01) the serum ALP levels but did not affect serum bilirubin levels.

### 2.4. Strawberry Extracts Effectively Regulate the Lipid Profile

Lipid profiles of control and treatment groups are depicted in Table 2. Diabetic rats had significantly higher (*p* ≤ 0.01) serum cholesterol (TC), high-density lipoprotein (HDL), low-density lipoprotein (LDL), very low-density lipoprotein (VLDL), and triglyceride (TG) levels than healthy rats. NIC-STZ rats receiving strawberry interventions hada significant decrease (*p* ≤ 0.01) in serum cholesterol (TC), LDL, VLDL, and triglyceride levels. Serum HDL level decreased significantly in aqueous-extract-treated animals and non-significantly in hydro-alcoholic strawberry-extract-treated animals. The serum HDL level increased in the animals treated with alcoholic strawberry extract.

### 2.5. Serum Creatinine Levels

Table 3 indicates the serum creatinine level at different time intervals in the control and treatment groups. In NIC-STZ rats, serum creatinine levels increased significantly (*p* ≤ 0.01) more than in the healthy control. Aqueous and hydro-alcoholic extracts lowered (*p* ≤ 0.01) serum creatinine levels.

### 2.6. Antioxidant Markers from Liver

Two antioxidant markers from liver namely malondialdehyde (MDA) and catalase (CAT) were selected since they are known to play an important role in neutralizing the effect of reactive oxygen species (ROS) thatare generated due to pathological conditions like diabetes [37,38]. The levels of these antioxidant markers in control and experimental animals are summarized in Table 4. High MDA and low CAT activity indicate oxidative stress in the livers of diabetic animals [39,40].

### 2.7. Strawberry Interventions Decreased MDA Content

Significantly increased MDA levels in NIC-STZ rats indicated lipid peroxidation in diabetic rats. The strawberry aqueous and hydro-alcoholic extracts significantly lowered the MDA content (*p* ≤ 0.01) as compared to the NIC-STZ control.

### 2.8. Strawberry Interventions Increased Catalase Activity in the Liver

The catalase activity significantly (*p* ≤ 0.05) decreased in STZ animals and significantly increased (*p* ≤ 0.01) after treatment with aqueous and alcoholic strawberry extracts. Treatment with alcoholic strawberry extracts significantly increased (*p* ≤ 0.01) catalase activity as against healthy and metformin MET-treated controls.

### 2.9. qRT-PCR Analysis of Fatty Acid Metabolism Genes, Inflammatory Markers, and Their Transcription Factors fromthe Liver

Gene expression analysis of three transcription factors regulating fatty acid metabolism genes, two inflammatory markers, and six genes involved in fatty acid metabolism revealed modulation in their expression patterns in the animals treated with the intervention. cDNA from liver tissues of control and experimental animals was prepared and qRT-PCR analysis was performed.

**Figure 1:**
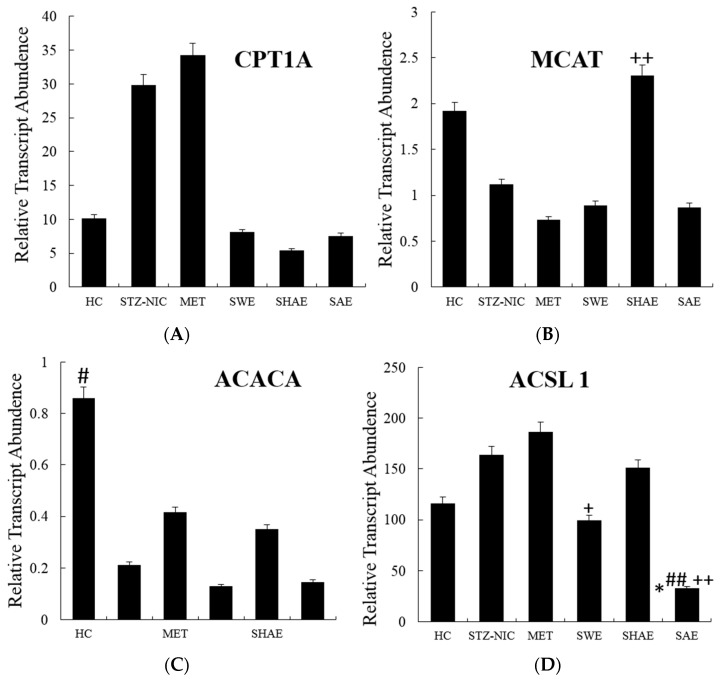

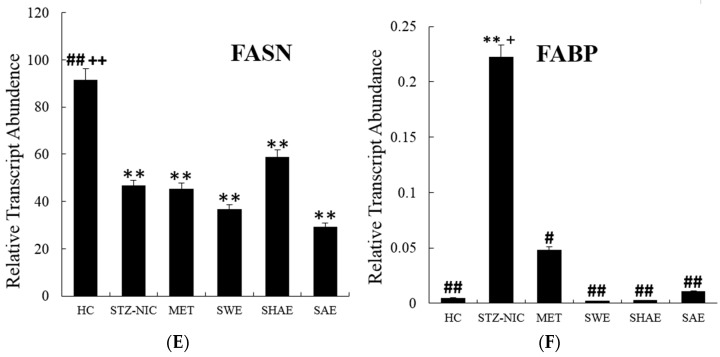
Expression profile of hepatic fatty acid metabolism genes. Animals from the SWE, SHAE, and SAE groups were treated daily with corresponding extracts at a dose of 2 g/kg body weight (Per os). **A**, **B**, **C**, **D**, **E** and **F** are expression profiles of the *CPT1A*, *MCAT*, *ACACA*, *ACSL1*, *FASN* and *FABP* genes, respectively. RNA was isolated from the animals’ livers and cDNA was prepared. cDNA was analyzed for the modulation of fatty acid metabolism genes. Results are represented as mean ± SE (*n* = 3 for each group and the reactions were performed in duplicate). Comparisons between the control group and experimental groups were performed by Dunnett’s multiple comparison test. (* *p* ≤ 0.05, ** *p* ≤ 0.01, when compared with thehealthy control group; ^#^ *p* ≤ 0.05, ^##^ *p* ≤ 0.01, when compared with the NIC-STZ control group; ^+^ *p* ≤ 0.05, ^++^ *p* ≤ 0.01, when compared with the MET-treated control group). HC: Healthy control, NIC-STZ: NIC-STZ treated control, MET: Metformin-treated control, SWE: Strawberry aqueous extract, SHAE: Strawberry hydro-alcoholic extract, SAE: Strawberry alcohol extract, *CPT1A*: Carnitine Palmitoyltransferase 1A, *MCAT*: *Malonyl-CoA*: ACP Acyltransferase, *ACACA*: Acetyl-CoA Carboxylase α, *ACSL1*: Acyl-CoA Synthetase Long-chain family member 1, *FASN*: Fatty Acid Synthase, *FABP*: Fatty Acid Binding Protein.

**Figure 2:**
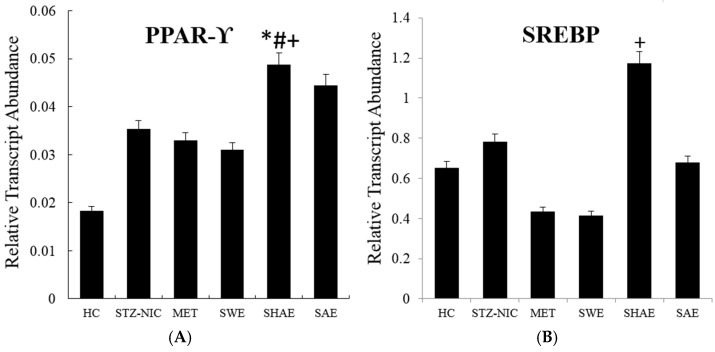

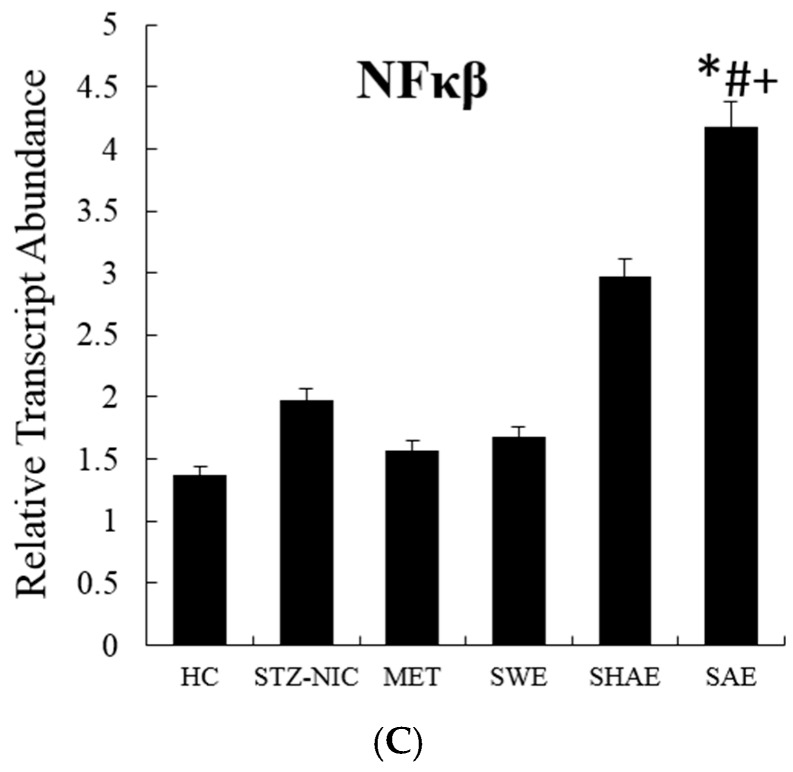
Expression profile of transcription factors from the liver. Animals from the SWE, SHAE, and SAE groups were treated daily with corresponding extracts at a dose of 2 g/kg body weight (Per os). **A**, **B** and **C** are expression profiles of the *PPAR-γ*, *SREBP* and *NF-κβ* genes, respectively. RNA was isolated from the animals’ livers and cDNA was prepared. cDNA was analyzed for the modulation of transcription factors of fatty acid metabolism genes. Results are represented as mean ± SE (*n* = 3 for each group and the reactions were performed in duplicate). Comparisons between the control group and each individual group were performed by Dunnett’s multiple comparison test. (* *p* ≤ 0.05, when compared with the healthy control group; ^#^ *p* ≤ 0.05, when compared with the NIC-STZ control group; ^+^ *p* ≤ 0.05, when compared with the MET-treated control group). HC: Healthy control, NIC-STZ: NIC-STZ treated control, MET: Metformin-treated control, SWE: Strawberry aqueous extract, SHAE: Strawberry hydro-alcoholic extract, SAE: Strawberry alcohol extract, *PPAR-γ*: Peroxisome Proliferator-Activated Receptor-γ, *SREBP*: Sterol Regulatory Element-Binding Proteins, *NF-κβ*: Nuclear Factor-κβ.

**Figure 3:**
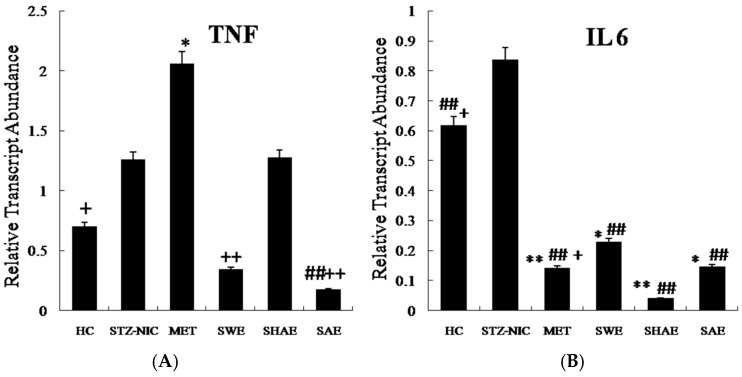
Expression profile of inflammatory markers from the liver. Animals from the SWE, SHAE, and SAE groups were treated daily with corresponding extracts at a dose of 2 g/kg body weight (Per os). **A** and **B** are expression profiles of the *TNF-α* and *Interleukin 6* genes, respectively. The total RNA was isolated from the animals’ livers and cDNA was prepared. The cDNA was analyzed for the modulation of inflammatory markers of fatty acid metabolism genes. Results are represented as mean ± SE (*n* = 3 for each group and the reactions were performed in duplicate). Comparisons between the control group and each individual group were performed by Dunnett’s multiple comparison test. (* *p* ≤ 0.05, ** *p* ≤ 0.01, when compared with the healthy control group; ^##^ *p* ≤ 0.01, when compared with the NIC-STZ control group; ^+^ *p* ≤ 0.05, ^++^ *p* ≤ 0.01, when compared with the MET-treated control group). HC: Healthy control, NIC-STZ: NIC-STZ treated control, MET: Metformin-treated control, SWE: Strawberry aqueous extract, SHAE: Strawberry hydro-alcoholic extract, SAE: Strawberry alcohol extract, *TNF-α*: Tumor Necrosis Factor-α, *IL6*: Interleukin 6.

Figure 1, Figure 2 and Figure 3 depict gene expression patterns of fatty acid metabolism, transcription factors, and inflammatory markers, respectively. Table S2 summarizes the efficiencies of qRT-PCR gene amplifications used for relative quantification of mRNA.

### 2.10. Fatty Acid Metabolism Genes Were Regulated by Treatment with Strawberry Extracts

The NIC-STZ rats showed a non-significant increase in the hepatic expression of the *CPT1A* gene, by 2.94 ± 0.53-fold as compared to the healthy control. The strawberry interventions effectively lowered the gene expression (Figure 1). The strawberry water, hydro-alcoholic, and alcohol extracts downregulated the hepatic expression of the *CPT1A* gene, by 3.68 ± 0.09-, 5.53 ± 0.8-, and 3.94 ± 0.8-fold, respectively, as compared to the NIC-STZ control, thus bringing it back to normal levels. In NIC-STZ rats, the hepatic expression of the *MCAT* gene was downregulated by 1.71 ± 0.8-fold as compared to the healthy control. The aqueous and alcoholic strawberry extracts did not show any effect on the regulation of the expression of *MCAT* gene but the hydro-alcoholic extract upregulated (*p* ≤ 0.05) the gene expression by 1.20 ± 0.24-fold as compared to the healthy control. In NIC-STZ rats, the hepatic expression of the *ACACA* was downregulated by 4.06 ± 0.99-fold as compared to the healthy control. The treatment of animals with strawberry extracts had no effect on the expression levels of *ACACA*. The expression of the hepatic *ACSL1* was non-significantly upregulated by 1.41 ± 0.15-fold in the NIC-STZ rats as compared to the healthy control. The animals receiving water extract showed downregulation of *ACSL1* by 1.65 ± 0.08-fold as compared to the NIC-STZ control, thus bringing it back to normal levels. In the strawberry alcohol extract, the expression was significantly decreased by 0.2 ± 0.06-fold below the NIC-STZ (*p* ≤ 0.01) control.

The NIC-STZ rats showed around two-fold downregulation (*p* ≤ 0.01) of the hepatic *FASN* gene as compared to the healthy control. The animals treated with alcoholic and water extracts showed downregulation, though insignificant, while those treated with hydro-alcoholic extracts showed an increase in the expression of hepatic *FASN*. In the NIC-STZ rats, the hepatic expression of *FABP* was upregulated (*p* ≤ 0.01), by 44.77 ± 1.26-fold as compared to the healthy control. The animals receiving strawberry interventions had significantly (*p* ≤ 0.01) low hepatic *FABP* expression as compared to the NIC-STZ- and MET-treated controls. Strawberry aqueous and hydro-alcoholic extracts were found to be more effective in normalizing the hepatic *FABP* expression.

### 2.11. Transcription Factors Expression Reverts Back to Near Normal after Intervention with Strawberry Extract

In the NIC-STZ rats, the hepatic expression of *PPAR-γ* was upregulated by 0.51 ± 0.13-fold as compared to the healthy control rats. The hepatic *PPAR-γ* expression was upregulated in animals receiving strawberry hydro-alcoholic (*p* ≤ 0.05) and alcoholic extracts as compared to all three control groups. The metformin-treated group and strawberry-water-extract-treated group showed downregulation of *PPAR-γ* expression as compared to the NIC-STZ control (Figure 2). Hepatic SREBP expression was non-significantly upregulated by 1.2 ± 0.13-fold in the NIC-STZ group overthe healthy control group. The metformin-treated control group and animals receiving water and alcoholic extracts had downregulation of the *SREBP* as compared to the NIC-STZ rats. The strawberry hydro-alcoholic extract, on the other hand, upregulated the expression by 1.5 ± 1.11-fold (Figure 2). The hepatic expression of *NF-κβ* was non-significantly upregulated in the NIC-STZ group as compared to the healthy control group. The strawberry water extract downregulated hepatic *NF-κβ* expression (Figure 2) and the expression levels were comparable to the MET-treated control. The strawberry hydro-alcoholic and alcohol extracts showed a significant increase in *NF-κβ* expression (*p* ≤ 0.05), by 1.50 ± 0.51- and 2.12 ± 0.75-fold, respectively, as compared to the NIC-STZ control.

### 2.12. Inflammatory Markers Were Effectively Downregulated by Strawberry Extract Interventions

In the NIC-STZ rats, both *TNF-α* and *IL6* were non-significantly upregulated by 1.63 ± 0.36- and 1.35 ± 0.24-fold, respectively, as compared to the healthy control. Hepatic expression of the *TNF-α* was downregulated non-significantly in the strawberry water extract, by 0.27 ± 0.009-fold as compared to the NIC-STZ control. Interestingly, the MET-treated control also showed an increase in the hepatic *TNF-α* gene expression by 1.63 ± 0.36-fold as compared to the NIC-STZ control. The strawberry water (*p* ≤ 0.01), hydro-alcoholic, and alcohol (*p* ≤ 0.01) extracts reduced the hepatic expression of hepatic *TNF-α* gene at varying levels. The *TNF-α* expression was decreased by 6.0 ± 0.18-, 0.61 ± 0.1- and 11.99 ± 0.02-fold, respectively, as compared to the MET-treated control (Figure 3).

The animals receiving the interventions showed significantly (*p* ≤ 0.01) downregulated expression of *IL6* as compared to the NIC-STZ rats (Figure 3). The strawberry water, hydro-alcoholic, and alcohol extracts showed decreased (*p* ≤ 0.01) hepatic expression of *IL6* by 3.64 ± 1.77-, 20.78 ± 0.16- and 5.74 ± 0.12-fold, respectively, as compared to the NIC-STZ control.

### 2.13. Histology Examination of Liver, Pancreas, Kidney, and Brain Tissue

Histology of four tissues, namely the liver, kidney, pancreas, and brain, were undertaken since these tissues are known to be affected by diabetes. For example, STZ kills the β cells of the pancreas and the effect of interventions can be detected at a histological level as well. Similarly, nephropathy is one of the secondary complications of uncontrolled hyperglycemia and the changes in the kidney can be visualized through histological examination.

**Figure 4:**
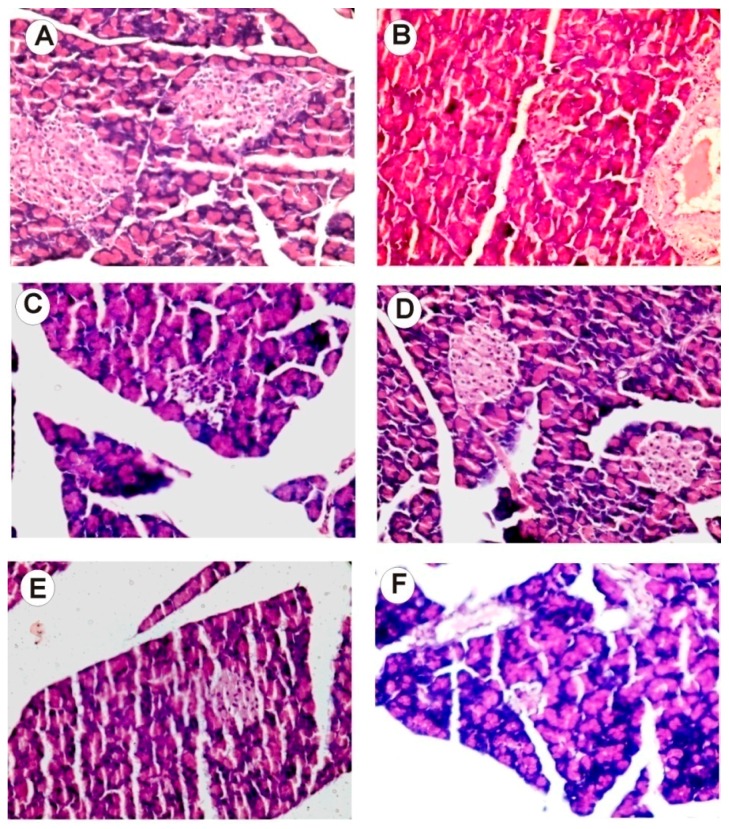

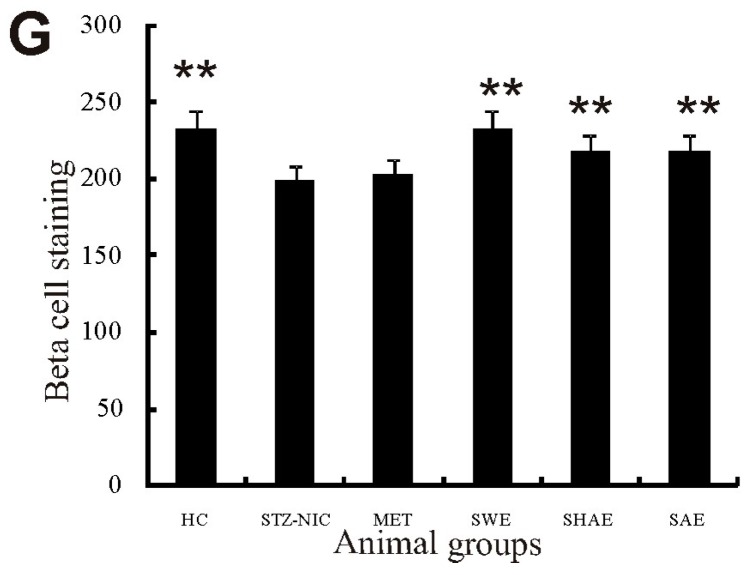
Hematoxylin- and eosin-stained cross sections of paraffin-embedded pancreas tissues of rats from control and experimental groups (40×). Pancreas cells from the healthy group showed normal architecture, with showed prominent Islets of Langerhans and β cells. In NIC-STZ rats the destruction of β cells was observed. The sections from the strawberry-water-extract-treated groups showed near-normal architecture. The aqueous strawberry extract exhibited comparatively more recovery in pancreatic architecture. Injury scores were calculated using the criteria described in the Methods (**G**). Graphical data are expressed as mean ± SE, and compared using Dunnett’s multiple comparison test against the NIC-STZ control (** *p* ≤ 0.01). (**A**) Healthy control; (**B**) NIC-STZ control; (**C**) MET-treated control; (**D**) strawberry water extract; (**E**) strawberry hydro-alcoholic extract; (**F**) strawberry alcoholic extract.

**Figure 5:**
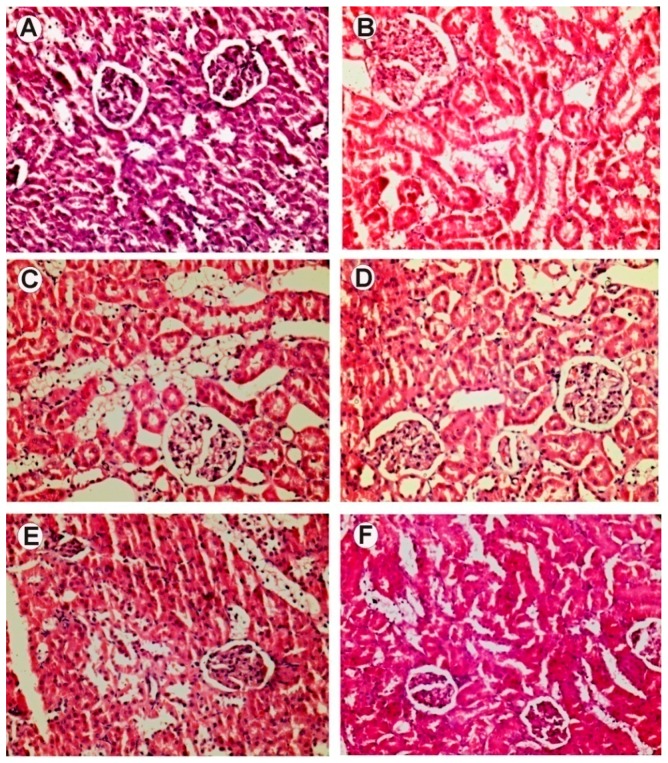

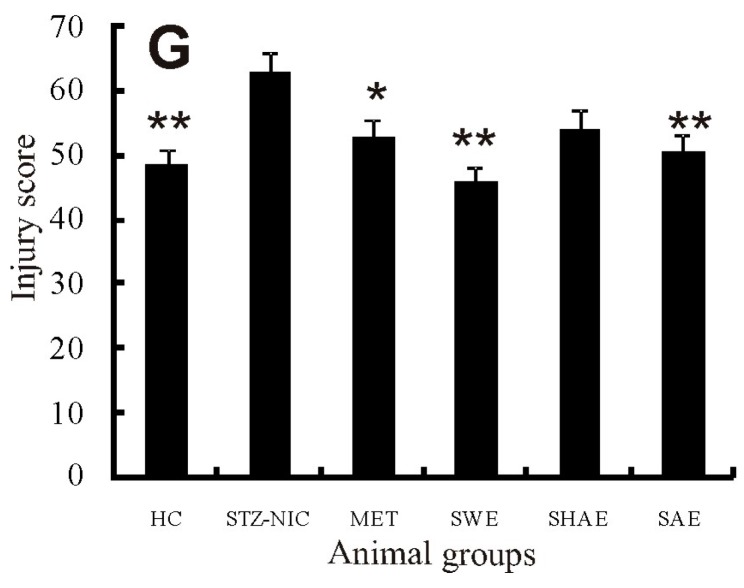
Hematoxylin- and eosin-stained cross sections of paraffin-embedded kidney tissues of rats from control and experimental groups (40×). In control rats, kidney and glomeruli appear to be normal (**A**). Tubules showed occasional vacuolated cells. NIC-STZ rats show tubules with vacuolated cells. Glomeruli show mesangial thickening (**B**). Strawberry interventions alter the histology of the kidney. Strawberry water extract (**D**) showed recovery of kidney architecture. Injury scores were calculated using the criteria described in the Methods (**G**). Graphical data expressed as mean ± SE, and compared using Dunnett’s multiple comparison test against the NIC-STZ control (* *p* ≤ 0.05 and ** *p* ≤ 0.01). The hydro-alcoholic and alcoholic extract showed changes (**E**,**F**). (**A**) Healthy control; (**B**) NIC-STZ control; (**C**) MET-treated control; (**D**) strawberry water extract; (**E**) strawberry hydro-alcoholic extract; (**F**) strawberry alcoholic extract.

Figure 4 depicts H&E-stained cross sections of paraffin-embedded pancreatic tissues of rats from control and experimental groups. In NIC-STZ-treated rats, pancreatic β cells were found to be damaged. The animals receiving the intervention of strawberry extract showed near normal Islets of Langerhans and β cells. Healthy rats had normal pancreatic architecture. Figure 5 shows H&E-stained cross sections of paraffin-embedded kidney tissues of rats from control and experimental groups. In control rats, kidney glomeruli appeared to be normal. In the NIC-STZ rats, the convoluted tubules had predominantly vacuolated cells and glomeruli showed mesangial thickening. Strawberry interventions significantly improved the kidney histology and showed maximum recovery from diabetic kidney damage. Histology of the brain and liver showed normal architecture in control and NIC-STZ rats (Material not intended for publicaiton) [41].

## 3. Discussion

The strawberry extracts significantly altered the lipid profile. Similar results were reported in a clinical study [42]. Strawberry seed oil lowered the activities of superoxide dismutase (SOD) and glutathione peroxidase (GPx) without any effect on the lipid profile in rats [43]. The strawberry extract was also effective in lowering serum TC and TG in animals fed on fructose-enriched diets [44]. Strawberry combined with yogurt was found to be effective against an abnormal lipid profile in mice [45]. The extracts of strawberry were extensively studied for their beneficial effects on cardiovascular diseases [42,46]. The antioxidant potential of fruit seems to play an important beneficial role in improving antioxidant defenses and thereby arresting the development of chronic diseases [47,48]. In our previous reports, we documented a high antioxidant activity of strawberry extracts [34,35,36].

The major phenolic compounds from strawberry-like quercetin, ellagic acid, and catechin, in their pure form, have been reported to have antidiabetic activities [49,50,51]. An extensive literature search indicated that there are very few previous reports on the effects of whole strawberry extracts or that of individual active molecules isolated from the extracts on diabetes [30,31,52]. To the best of the authors’ knowledge, the present report is the first comprehensive report investigating the effect of whole fruit extracts of strawberry on streptozotocin-induced diabetes at biochemical, molecular, and histological levels.

**Figure 6:**
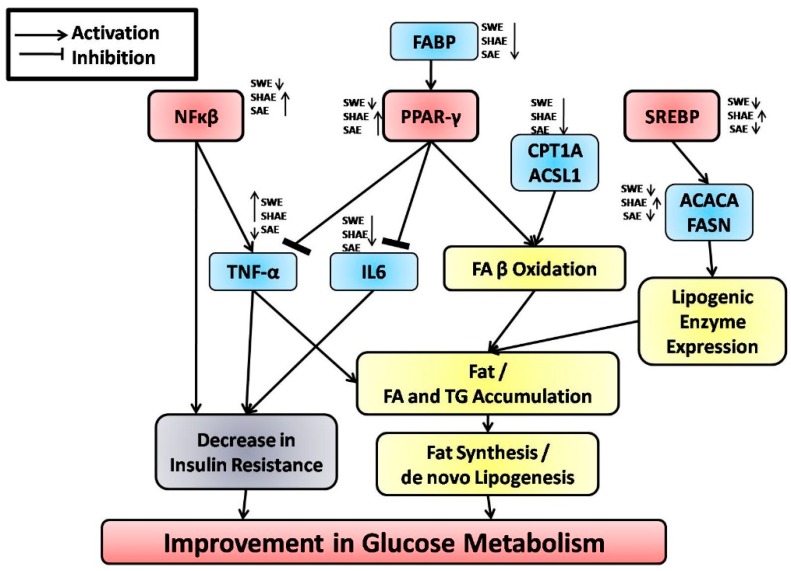
Network diagram representing hepatic transcription factors and genes and their effects on lipid metabolism and the effect of the strawberry extract on their hepatic expression as observed in this study. *CPT1A*: Carnitine Palmitoyltransferase 1A, *MCAT*: *Malonyl-CoA:* ACP Acyltransferase, *ACACA*: Acetyl-CoA Carboxylase α, *ACSL1*: Acyl-CoA Synthetase Long-chain family member 1, *FASN*: Fatty Acid Synthase, *FABP*: Fatty Acid Binding Protein, *PPAR-γ*: Peroxisome Proliferator-Activated Receptor-γ, *SREBP*: Sterol Regulatory Element-Binding Proteins, *NF-κβ*: Nuclear Factor-κβ, *TNF-α*: Tumor Necrosis Factor-α, *IL6*: Interleukin 6. Upward and downward arrows near strawberry water extract (SWE), strawberry hydroalcoholic extract (SHAE) and strawberry alcoholic extract (SAE) indicates upregulation and downregulation of the particular gene, respectively.

The model for the probable mechanism of action of the strawberry extract is presented in Figure 6. The transcription factor, *PPAR-γ*, a member of the nuclear receptor family of PPARs, plays a key role in maintaining carbohydrate and lipid homeostasis [53]. The activation of *PPAR-γ* has been shown to stimulate β-oxidation of fatty acids, thereby reducing the serum TG level [54]. In our experiments, strawberry aqueous and hydro-alcoholic extracts significantly upregulate the hepatic expression of *PPAR-γ*, which may be one of the factors for lowering the TG levels in serum. It also significantly downregulates the hepatic *TNF-α* and *IL6* expression, thereby lowering serum TG levels and inhibiting lipogenesis, besides having an anti-inflammatory effect.

*SREBP*s are transcription factors involved in the regulation of fatty acid and cholesterol metabolism in the liver [55]. Shimomura et al. [56] reported increased expression of lipogenic genes, *acetyl-CoA carboxylase* (*ACACA*), and *fatty acid synthase* (*FASN*) in diabetic mice following the overexpression of *SREBP-1*. In our experiments, hepatic expression of *SREBP* and consequently, *CPT1A*, *ACACA*, *ACSL1*, *FASN*, and *FABP* genes was also found to be downregulated. Thus, downregulation of *SREBP-1* in the liver has a therapeutic value in treating diabetic hepatic steatosis and carbohydrate-induced hypertriglyceridemia [57]. From our data, it is clear that the strawberry interventions effectively regulate the transcription factors involved in the pathophysiology of diabetes, thereby modulating the genes associated with inflammation and fatty acid metabolism.

Previous studies have indicated a key role of *NF-κβ* in the pathogenesis of insulin resistance and type 2 diabetes mellitus [58,59,60]. Overexpression of *IKK-β* in the liver, which causes sustained activation of *NF-κβ*, as seen in chronic liver inflammation, mimics a high-fat diet or obesity-induced insulin resistance. Conversely, attenuation of *NF-κβ* activation in the liver diminishes the expression of *NF-κβ*-dependent genes and also reverses the phenotypes of type 2 diabetes as well [60]. Systemic neutralization of *IL6* also exhibits a significant improvement in insulin resistance in mice [61]. Administration of a specific inhibitor of *IL1* signaling has been shown to ameliorate inflammation-induced hyperglycemia [59]. These results clearly suggest that *NF-κβ* and its target genes, such as *TNF-α*, *IL1*, and *IL6*, are critical in the development of inflammation and insulin resistance. In the present study, hepatic *NF-κβ* was downregulated and subsequently *TNF-α* and *IL6* also displayed a similar trend in their expression levels.

In fatty acid metabolism, ACACA catalyzes the rate-limiting reaction in the biogenesis of long-chain fatty acids and acts as a biotin carboxyl carrier protein, biotin carboxylase, and carboxyltransferase [61]. In normal rats, an increase in *ACACA* expression leads to a decrease in *CPT1A* gene expression, thus preventing fatty acid oxidation. However, under hyperglycemic conditions, fatty acid oxidation results in the release of ketone bodies and free fatty acids in the serum [62]. ACACA catalyzes the formation of malonyl-CoA with multiple fates [63]. Malonyl-CoA thus formed and acetyl-CoA are utilized by FASN to form long-chain fatty acids. The *FASN* gene catalyzes the formation of long-chain fatty acids from acetyl-CoA, malonyl-CoA, and NADPH [64]. At the transcription level, *FASN* is mainly regulated by nutrients and hormones [65]. Glucose (via *ChREBP*) and insulin (via *SREBP-1c*) increase *FASN* activity, whereas glucagon and saturated/polyunsaturated fatty acids decrease it [66]. Our results are in accordance with these findings. In diseased conditions, the increased *FASN* expression led to an increase in long-chain fatty acids synthesis and their transport to mitochondria. Though fatty acid synthesis was brought back to near normal (SWE and SAE), the fatty acid transport (expression of *CPT1A*) was comparable to the healthy control.

After synthesis, long-chain fatty acids are targeted to various organs [67,68]. FABP binds with free fatty acids and their coenzyme A derivatives, bilirubin, and some other small molecules in the cytoplasm. It is also involved in intracellular lipid transport, i.e., fatty acid uptake, transport, and metabolism [67]. In the NIC-STZ control, *FABP* expression increased significantly, which may release/transport free fatty acids across the membrane. In all three extracts, the expression was comparable to the healthy control, indicating the effect of extracts on *FABP* regulation. The excess fatty acids are utilized for the generation of new acyl-CoA esters. ACSL1 is involved in the breakdown of the complex fatty acids via production of long-chain fatty acyl-CoA esters, which affects protein transport, enzyme activation, protein acylation, cell signaling, and transcription factors [69]. In the intervention groups, there is an upregulation of fatty acid synthesis and simultaneously near-normal transport of fatty acids in mitochondria.

In the diabetic condition, expression of *CPT1A* was increased. CPT1A is involved in the transfer of the acyl group of long-chain fatty acyl-CoA and conjugates onto carnitine [70]. It is known that *CPT1A* facilitates the uptake of fatty acids by mitochondria and consequent β oxidation [70]. All three strawberry extracts downregulated the *CPT1A* expression, bringing it back to a normal level. This is a rate-limiting enzyme in mitochondrial fatty acid oxidation and the concomitant release of free fatty acids. This may be the probable reason for the low serum TG levels observed with strawberry extract treatment in the present study.

*NF-κβ* regulates the expression of inflammatory markers such as *IL6* and *TNF-α*. All three extracts significantly downregulated *IL6* expression. Histological examination also revealed the regeneration/protection of β-cells in the pancreas. The combined effect of improving insulin sensitivity by *IL6* regulation and regeneration/protection of β-cells may be the reason for glucose regulation in treated groups. Tumor necrosis factorα (*TNF-α*) has been demonstrated to regulate and interfere with energy metabolism, especially lipid homeostasis [71,72]. These include reduction of HDL-cholesterol, increase of LDL-cholesterol, and elevated expression of cholesterogenic genes [72]. In our study, the MET-treated control showed elevated LDL-cholesterol with a simultaneous decrease in HDL-cholesterol. In the strawberry-extract-treated groups, the LDL-cholesterol level decreased significantly as compared to NIC-STZ mice. These results are in accordance with previous studies [71,72].

In strawberry-aqueous-extract-treated animals, *CPT1A*, *ACSL1*, and *FABP*, the three transcription factors (*SREBP*, *PPAR-γ*, and *NF-κβ*), and two inflammatory markers (*IL6* and *TNF-α*) were found to be upregulated in diabetic animals as compared to healthy controls. The animals receiving the intervention of strawberry water extract showed downregulation of *MCAT*, *FASN*, and *ACACA* as compared to healthy and negative controls. The strawberry water extract normalized *FABP*, *ACSL1A*, *CPT1A*, and *NF-κβ* expression. This may be the reason behind decreased serum TG, TC, and LDL levels and normalizing the serum HDL levels. The extract also downregulated *TNF-α*, *IL6*, and *SREBP* genes as compared to negative and healthy controls. Hence, the extract has an effect on the binding of fatty acids (*FABP*) and the activation of long-chain fatty acids (*ACSL1A*) for the synthesis of cellular lipids, transport through carnitine shuttle (*CPT1A*), and degradation via mitochondrial β-oxidation. The extract has an effect on transcription factors as well as their regulatory genes. In the present study, *SREBP*, *ACSL1*, and *CPT1A* were found to be downregulated by strawberry water extract. Hepatic *IL6* and *TNF-α* were downregulated and *NF-κβ* was near normal as compared to negative as well as healthy controls. The *PPAR-γ* gene was also downregulated in animals treated with strawberry extracts as compared with the negative control. *NF-κβ* is known to regulate the expression of inflammatory markers like *IL6* and *TNF-α* [59], which is evident from the results obtained in the present study. The animals treated with strawberry water extract exhibited near-normal pancreatic histology with probable regenerative/protective effect on β-cells. Besides normal serum creatinine levels, the histological examination also showed near-normal kidney histology. The strawberry extract thus controlled/inhibited diabetes-associated complications like kidney damage.

In the negative control of strawberry hydro-alcoholic extracts (SHAE)-treated animals, *ACACA* and *FASN* expression decreased as compared to healthy controls, which indicates decreased lipogenesis. The increase in hepatic *ACACA* (malonyl-CoA levels) expression corresponds to a decrease in blood ketone bodies, which in turn activates *PPAR-γ* [73]. Activation of *PPAR-γ* has a negative effect on *TNF-α* and *IL6* expression. At the same time, *TNF-α* is positively regulated by transcription factor *NF-κβ*, which is upregulated by the treatment. This may be the probable reason behind the overexpression of the *TNF-α* gene. Activation of *PPAR-γ* induces β-oxidation in hepatic mitochondria [74]. In short, with the treatment of the extract, lipogenesis increased, but *FABP* and *CPT1A* expression were brought to near-normal levels. *MCAT* gene expression was found to be increased as compared to the negative control but *FABP* and *CPT1A* expressions were comparable to healthy animals. The increase in hepatic *SREBP* expression indicated the downregulation of the AMPK pathway. Increased expression of *SREBP* was in turn reflected in an increase in the expression of *ACACA* and *FASN*.

In strawberry-alcoholic-extract-treated animals, expression levels of *ACACA*, *ACSL1*, *CPT1A*, *FABP*, and *FASN* were found to be decreased after the intervention as compared to the negative control. The decrease in *SREBP* expression was normalized by the intervention of strawberry alcohol extract. This may lead to activation of the AMPK pathway. Increased hepatic expression of the *PPAR-γ* gene after the intervention leads to a decrease in the expression of *IL6* and *TNF-α*.

We demonstrate that the strawberry extracts act on multiple cellular targets. Normalization of expression of transcription factors (*PPAR-γ*, *SREBP*, and *NF-κβ*) was found to bring inflammation and fatty acid metabolism to near-normal states. Downregulation of *SREBP* may have a direct effect on the AMPK pathway, thereby reducing serum glucose, which may delay the diabetic complications associated with consistent hyperglycemia.

## 4. Materials and Methods

### 4.1. Chemicals

All chemicals used were of analytical grade. Bovine serum albumin (BSA) was obtained from Fluka Chemie (Buchs, Switzerland). Streptozotocin, butylated hydroxytoluene (BHT), thiobarbituric acid (TBA), trichloroacetic acid (TCA), 1,3,3,3-tetra-ethoxy propane, and hydrogen peroxide (H_2_O_2_) were purchased from Sigma-Aldrich (St. Louis, MO, USA). Citric acid and trisodium citrate were procured from Merck (Darmstadt, Germany). Sodium phosphate dibasic (Na_2_HPO_4_), sodium phosphate monobasic (NaH_2_PO_4_), and ammonium molybdate were obtained from SRL (Mumbai, India).

### 4.2. Collection of Strawberry Fruit

Fruits of *Fragaria x ananassa* Duch. cv. *Sweet Charlie* were harvested from a commercial plantation located at Mahabaleshwar, Maharashtra, India (17.9217° N, 73.6556° E), 1438 m above mean sea level (MSL). The fruits were harvested in the early morning. The fruits were collected directly from the field. The harvested fruits were snap frozen in liquid nitrogen and stored at −80 °C until analyzed.

### 4.3. Preparation of Extract

Fruits were crushed in the desired solvent, viz. water, water: ethanol (50:50, *v*/*v*), or ethanol. Fruits (100 gm) were crushed in 100 mL of each solvent. The homogenate was filtered through a muslin cloth and the solvent was allowed to evaporate for 3 h at room temperature. The extracts were prepared fresh every day for animal treatment.

### 4.4. Experimental Animals

The studies were carried out as per the Committee for the Purpose of Control And Supervision of Experiments on Animals (CPCSEA) guidelines and after approval of the Institutional Animal Ethics Committee (Ref. No.: BVDUMC/443/2012–2013, Permission date: 09.03.13). Three-month-old male albino Wistar rats weighing between 150 and 200 g were procured from the institutional animal house. They were acclimatized to animal house facilities for seven days and maintained under standard conditions (temperature 25 ± 2 °C, 12-h light: 12-h dark cycle) throughout the experiment. The animals were fed with a standard pellet diet (Nutrivet life science, Pune, India) and water was supplied adlibitum.

### 4.5. Experimental Induction of Diabetes

Rats were randomly selected and divided into six groups of six animals each. Diabetes was induced by intraperitoneal injection of streptozotocin (65 mg/kg body weight, intraperitoneal) suspended in ice-cold 100 mM citrate buffer (pH 4.5). Nicotinamide (110 mg/kg b.w., i.p.) was administered 30 min before STZ induction. The dose of extracts was finalized on the basis of earlier studies carried out in the laboratory. The tabular protocol is depicted in Table S3. The treatment protocol was as follows: group I: healthy Control (*n* = 6); received food and water normally for fourweeks; group II: NIC-STZ Control (*n* = 6); administered streptozotocin (65 mg/kg b.w./day, i.p.); group III: MET-treated control (*n* = 6); administered streptozotocin (65 mg/kg b.w./day, i.p.) + Metformin (200 mg/kg b.w./day, p.o.) daily, for four weeks; group IV: treatment group 1 (*n* = 6); administered streptozotocin (65 mg/kg b.w./day, i.p.) + aqueous (water) strawberry extract (2 g/kg b.w./day, p.o.) daily, for four weeks; group V: Treatment group 2 (*n* = 6); administered streptozotocin (65 mg/kg b.w./day, i.p.) + hydro-alcoholic strawberry extract (2 g/kg b.w./day, p.o.) daily, for four weeks; group VI: treatment group 3 (*n* = 6); administered streptozotocin (65 mg/kg b.w./day, i.p.) + alcoholic strawberry extract (2 g/kg b.w./day, p.o.) daily, for four weeks.

### 4.6. Collection of Blood and Tissues

The animals were observed daily for any signs of discomfort and/or infection. Retro-orbital blood was collected on the 30th, 36th, and 43rd day after initiation of the experiment. It was allowed to clot at room temperature for 30 min and serum was collected after centrifugation (Eppendorf centrifuge 5415D, Eppendorf AG, Hamburg, Germany) at 2000 rpm for 15 min. Serum was analyzed for different biochemical parameters. After four weeks (from the 23rd to the 49th day) of continuous treatment, animals were fasted overnight. The animals were humanely sacrificed the next day (50th day) under light ether anesthesia and blood was collected by cardiac puncture. Liver, brain, pancreas, and kidney tissues were excised immediately, blotted of blood, and parts were washed in saline, weighed, and stored in 10% neutral buffered formalin for histological examination, while the remaining tissue was stored in liquid nitrogen for molecular and biochemical analysis.

### 4.7. Blood Biochemistry from Serum

Serum was subjected to glucose estimation, liver function tests, and lipid profile. Marker enzymes of liver damage (serum glutamic oxaloacetic transaminase (SGOT), serum glutamic pyruvic transaminase (SGPT) and alkaline phosphatase (ALP)), total bilirubin, total cholesterol, HDL cholesterol, and total triglycerides were estimated using commercial kits (Coral Clinical System, Goa, India). LDL-cholesterol (mg/dL) was estimated by using the formula: (Total Cholesterol × HDL Cholesterol) × triglycerides/5 and VLDL cholesterol was estimated by using the formula: Triglycerides/5 [75,76].

### 4.8. Antioxidant Enzyme Assays from Liver

Part of the liver tissue was homogenized by a mortar and pestle in 20 mM tris buffer (pH 7.4) containing 5 mM butylated hydroxytoluene (BHT). Homogenate was then centrifuged at 12,000× *g* at 4 °C for 10 min (Eppendorf centrifuge 5810R, Eppendorf AG, Hamburg, Germany). The supernatant was stored at −80 °C and used to determine total protein content, malondialdehyde (MDA) and catalase activity.

### 4.9. Protein Estimation

Protein estimation was done by the Bradford method [77]. Bovine serum albumin (BSA) was used as the standard. The protein concentration was expressed in μg/μL mg liver tissue.

### 4.10. Estimation of Malondialdehyde (MDA)

Lipid peroxidation, an indicator of tissue injury induced by reactive oxygen species, was measured as thiobarbituric acid reactive substance (TBARS). The amount of tissue TBARS was measured by the thiobarbituric acid assay (TBA), as previously described by Buege and Aust [78]. Briefly, 500 μL of tissue homogenates were mixed with 2 mL of TBA reagent containing 0.375% TBA, 15% trichloroacetic acid, and 0.25 N HCl. The reaction contents were mixed and the reaction was boiled for 15 min, cooled, and centrifuged. The absorbance of the supernatants was spectrophotometrically measured at 532 nm (ELISA plate reader, BIO-RAD, Mumbai, India). A TBARS concentration was calculated from the standard curve prepared with 1,3,3,3-tetra-ethoxypropane as a standard. The results were expressed as µmol/g protein.

### 4.11. Estimation of Catalase (CAT)

Catalase was estimated by the method described by Goth et al. [79]. Briefly, 200 μL of tissue homogenates was incubated with 1 mL of the substrate (65 μM H_2_O_2_ in 60 mM sodium-potassium phosphate buffer, pH 7.4) at 37 °C for 60 s. The reaction was stopped with 1 mL of 32.4 mM ammonium molybdate ((NH_4_)_6_Mo_7_O_24_·4H_2_O) and the yellow complex of molybdate and hydrogen peroxide was measured at 405 nm (ELISA plate reader, BIO-RAD) against blank 3.

Serum catalase activity (kU/L) = [(A_(sample)_ − A_(blank 1)_)/(A_(blank 2)_ − A_(blank 3)_)]× 271.

Blank 1 contained 1 mL substrate, 1 mL molybdate, and a 200 μL supernatant; blank 2 contained 1 mL substrate, 1 mL molybdate, and 0.2 mL buffer; blank 3 contained 1.2 mL buffer and 1 mL molybdate. One unit of catalase activity was defined as the activity that decomposes 1 pmol of hydrogen peroxide/min under these conditions.

### 4.12. Expression Study from Liver cDNA

The modulation of expression of genes was studied from the liver. For qRT-PCR analysis, the total RNA from the liver tissues was extracted using TRIZOL reagent (Invitrogen, Carlsbad, CA, USA). The quality of the isolated RNA was determined using agarose gel electrophoresis. The RNA quantification was done on an ND-1000 UV spectrophotometer (Nanodrop Technologies, Wilmington, DE, USA). The cDNA was synthesized from 4 ng of total RNA using the SuperScript first-strand synthesis system for quantitative real-time PCR (Invitrogen).

Three transcription factors (Sterol Regulatory Element Binding Transcription factor (SREBP), Nuclear Factor-κβ (*NF-κβ*) and Peroxisome Proliferator-Activated Receptor Gamma (PPAR-γ)) that regulate fatty acid metabolism genes (Fatty Acid Binding Protein (FABP), Fatty Acid Synthase (FASN), Carnitine palmitoyl transferase 1A (CPT1A), Acyl-CoA Synthetase Long-chain family member 1 (ACSL1), Acetyl-CoA Carboxylase α (ACACA) and Malonyl-CoA: ACP Acyltransferase (MCAT)), along with two inflammatory markers (Interleukin 6 (IL6) and Tumor Necrosis Factor-α (TNF-α)), were selected for the analysis. The primer sequences of selected genes are depicted in Table S4. Expression analysis of all genes was performed by qRT-PCR using the iCycler system. The following PCR program was run: initial denaturation step at 94 °C for 10 min; 40 cycles of denaturation at 94 °C for 3 s; annealing at 60 °C for 30 s and extension at 95 °C for 15 s; final extension step at 60 °C for 0.15 s. Three biological replicates were analyzed. Each reaction was performed at least in duplicate and the corresponding *C*t values were normalized using the *C*_t_ value corresponding to the glyceraldehyde-3-phosphate dehydrogenase (*GAPDH*) gene (as a housekeeping gene). All these values were then used to determine the relative change in gene expression levels of the target genes with respect to the control.

### 4.13. Histological Examination and Estimation of Staining Intensity

Paraffin-embedded liver, pancreas, kidney, and brain tissues were cut to 4 μm thickness and stained with Hematoxylin and Eosin. The slides were examined under a microscope and photographed.

Staining intensity was calculated as described by Campbell et al. [80] with some modifications. Briefly, six images from each animal section were calibrated and adjusted using image J (National Institutes of Health, Bethesda, MD, USA) until only the stain deposits were visible without detectable background. The intensity of the image was calculated from the software. Image analysis resulted in the stained total area in pixels squared. Densitometric values for the total area for all animal sections in each group were used to determine the average renal injury score and β cell staining in the pancreas.

### 4.14. Statistical Analysis

The data were subjected to statistical analysis using Graph Pad Instat (Version 3, GraphPad Software Inc., San Digeo, CA, USA). Results are presented as Mean ± Standard Error (SE). Dunnett Multiple Comparison Test and one-way Analysis of Variance (ANOVA) were performed to estimate the statistical significance between groups. For qRT-PCR analysis, Excel templates provided by SA biosciences were used along with their web analysis tool located at http://pcrdataanalysis.sabiosciences.com/pcr/arrayanalysis.php.

## 5. Conclusions

In summary, the biochemical and molecular studies in chemically induced diabetic rats demonstrate that the strawberry extract, a concentrated source of polyphenolic flavonoids, fiber, and phytosterols, is a potential dietary fruit supplement that may be effective in the management of type 2 diabetes and its associated complications. The protective and therapeutic effects of strawberry extracts against liver and kidney injury in diabetes are worth investigating further.

## Figures and Tables

**Table 1:** Serum glucose level (mg/dL) of control and experimental animals. Strawberry water extract (SWE), strawberry hydro-alcoholic extract (SHAE), and strawberry alcohol extract (SAE) were treated with 2 g/kg body weight (Per os).

Groups	30th Day	36th Day	43rd Day	50th Day
Healthy Control	111.47 ± 7.02 **	129.38 ± 3.02 **	117.48 ± 11.62 **	86.08 ± 7.70 **
NIC-STZ Control	353.60 ± 15.87	486.46 ± 45.69	664.19 ± 21.48	708.47 ± 12.66
MET-treated Control	578.98 ± 28.68 **	551.57 ± 6.39 ^ns^	555.92 ± 6.99 **	411.69 ± 11.02 **
SWE	531.01 ± 32.35 **	473.17 ± 31.53 ^ns^	504.18 ± 30.61 **	371.16 ± 15.41 **
SHAE	537.04 ± 1.1.6 **	589.57 ± 12.92 *	482.54 ± 2.86 **	400.00 ± 16.74 **
SAE	525.27 ± 2.45 **	518.12 ± 2.45 ^ns^	421.67 ± 3.58 **	375.71 ± 2.24 **

Results are a mean of three replicates and are represented as mean ± SE. * *p* ≤ 0.05; ** *p* ≤ 0.01 as compared to NIC-STZ control group (Dunnett Multiple Comparisons Test). NIC-STZ: nicotinamide-streptozotocin, MET: Metformin, ns: Non-significant.

**Table 2:** Levels of liver marker enzymes, bilirubin, and lipid profile of control and experimental animals. Strawberry water extract (SWE), strawberry hydro-alcoholic extract (SHAE), and strawberry alcohol extract (SAE) were treated with 2 g/kg body weight (Per os).

Group	Liver Function Tests				Lipid Profile				
	SGOT (U/mL)	SGPT (U/mL)	ALP (U/mL)	BIL (mg/dL)	TC (mg/dL)	HDL (mg/dL)	LDL (mg/dL)	VLDL (mg/dL)	TG (mg/dL)
Healthy Control	38.43 ± 4.03 **	17.32 ± 1.16 **	23.39 ± 0.56 **	0.58 ± 0.05 **	62.24 ± 2.37 **	28.73 ± 3.25 **	27.4 ± 2.29 **	6.13 ± 0.46 **	30.62 ± 2.30 **
NIC-STZ Control	346.94 ± 5.97	83.54 ± 2.20	134.91 ± 2.02	1.78 ± 0.05	213.69 ± 2.49	55.99 ± 6.33	138.65 ± 7.70	19.01 ± 0.34	95.12 ± 1.69
MET-treated Control	261.48 ± 6.56 **	82.76 ± 2.82 ^ns^	127.8 ± 4.32 ^ns^	2.51 ± 0.11 ^ns^	174.58 ± 3.29 **	23.59 ± 3.91 **	137.82 ± 5.37 ^ns^	13.19 ± 0.54 **	65.96 ± 2.70 **
SWE	104.54 ± 7.98 **	25.83 ± 1.38 **	119.66 ± 4.29 **	1.14 ± 0.08 ^ns^	125.17 ± 4.45 **	32.06 ± 3.02 **	81.77 ± 5.29 **	11.34 ± 1.72 **	56.71 ± 8.57 **
SHAE	181.84 ± 4.39 **	53.7 ± 5.90 **	109.4 ± 2.23 **	2.44 ± 0.09 ^ns^	98.59 ± 2.93 **	41.5 ± 4.45 ^ns^	45.53 ± 5.57 **	11.56 ± 1.58 **	57.86 ± 7.88 **
SAE	347.73 ± 0.97 ^ns^	49 ± 4.41 **	146.8 ± 0.03 *	3.96 ± 0.50 **	127.13 ± 0.45 **	58.23 ± 1.76 ^ns^	51.57 ± 1.35 **	17.33 ± 0.08 ^ns^	86.6 ± 0.39 ^ns^

Results are a mean of three replicates and are represented as mean ± SE. * *p* ≤ 0.05; ** *p* ≤ 0.01 as compared to NIC-STZ control group (Dunnett Multiple Comparisons Test). SGOT: Serum glutamic oxaloacetic transaminase; SGPT: Serum glutamic pyruvic transaminase; ALP: Alkaline phosphatase; BIL: Total bilirubin; TC: Total Cholesterol; HDL: High-density lipoprotein; LDL: Low-density lipoprotein; VLDL: Very low-density lipoprotein; TG: Triglycerides. NIC-STZ: nicotinamide-streptozotocin, MET: Metformin, ns: Non-significant.

**Table 3:** Creatinine levels (% mg) of control and experimental animals. Strawberry water extract (SWE), strawberry hydro-alcoholic extract (SHAE), and strawberry alcohol extract (SAE) were treated with 2 g/kg body weight (Per os).

Group	% mg
Healthy Control	1.38 ± 0.11 **
NIC-STZ Control	3.03 ± 0.10
MET-treated Control	1.86 ± 0.20 **
SWE	1.73 ± 0.07 **
SHAE	1.74 ± 0.07 **
SAE	2.97 ± 0.01 ^ns^

Results are mean of three replicates and are represented as mean ± SE. ** *p* ≤ 0.01 as compared to NIC-STZ control group (Dunnett Multiple Comparisons Test). NIC-STZ: nicotinamide-streptozotocin, MET: Metformin, ns: Non-significant.

**Table 4:** Antioxidant enzymes from the liver. Strawberry water extract (SWE), strawberry hydro-alcoholic extract (SHAE), and strawberry alcohol extract (SAE) were treated with 2 g/kg body weight (Per os) respectively.

Groups	MDA (μM/g Protein)	CAT (kU/L) × 10^2^
Healthy Control	3.147 ± 0.06 **	24.05 ± 0.37 *
NIC-STZ Control	8.628 ± 1.54 ^##,++^	19.09 ± 0.16 ^+^
MET-treated Control	4.622 ± 0.30 **	24.76 ± 2.36 *
SWE	5.95 ± 0.95 **	27.66 ± 1.10 **
SHAE	3.59 ± 0.62 **	22.05 ± 1.77
SAE	7.25 ± 0.03 ^##^	37.70 ± 0.12 ^++,^**

Results are mean of three replicates and are represented as mean ± SE. ^##^ *p* < 0.01, when compared with the healthy control group; * *p* < 0.05, ** *p* < 0.01, when compared with the NIC-STZ control group; ^+^ *p* < 0.05, ^++^ *p* < 0.01 when compared with the MET-treated control group (Dunnett Multiple Comparisons Test). NIC-STZ: nicotinamide-streptozotocin, MET: Metformin, kU/L: Katal Unite per liter.

## Supplementary Materials

Supplementary materials can be found at www.mdpi.com/1422-0067/18/1/124/s1.
