# Supplementary material for: Antidiabetic, Lipid Normalizing, and Nephroprotective Actions of the Strawberry: A Potent Supplementary Fruit
Source: Int J Mol Sci. 2017 Jan 11;18(1):124. doi: 10.3390/ijms18010124 (PMC5297758; doi:10.3390/ijms18010124)
Supplement: Supplementary file 1 [file ijms-18-00124-s001.pdf]

Supplementary Materials: Antidiabetic, Lipid Normalizing, and Nephroprotective Actions of the Strawberry: A Potent Supplementary Fruit

Pallavi Mandave, Suresh Khadke, Manjiri Karandikar, Vijaya Pandit, Prabhakar Ranjekar, Aniket Kuvalekar and Nitin Mantri

Table S1. Animals' food and water intake during interventions.

Groups	Feed Intake (gm)	Water Intake (mL)
Healthy control	22.3 ± 1.24 *	50.24 ± 1.93 **
NIC-STZ control	44.1 ± 0.52	138.6 ± 1.64
MET-treated group	42.4 ± 9.51	94.19 ± 0.74 **
SWE	37.9 ± 9.56 *	87.13 ± 0.63 ***
SHAE	26.7 ± 0.28 **	80 ± 3.24 **
SAE	59.8 ± 1.144 **	70 ± 0.45 **

Results are represented as mean ± SE. gm/rat/day ($n = 6$); * $p \leq 0.05$; ** $p \leq 0.01$; *** $p \leq 0.001$ as compared to the negative control group (Dunnett Multiple Comparisons Test).

Table S2. The efficiency of qRT-PCR for relative quantification of mRNA. Efficiency is calculated from the slope of the curve as $E = 10(-1/\text{slope})^{-1}$.

Sr. No.	Target Genes	Symbol	Efficiency
	**Fatty Acid Metabolism Genes**		
1	Carnitine Palmitoyltransferase 1A	CPT 1A	121.22
2	Malonyl CoA: ACP Acyltransferase	MCAT	110.17
3	Acetyl-CoA Carboxylase Alpha	ACACA	110.17
4	acyl-CoA Synthetase Long-chain family member 1	ACSL 1	110.17
5	Fatty Acid Synthase	FASN	93.06
6	Fatty Acid Binding Protein	FABP	101.30
	**Transcription Factors**		
1	Peroxisome Proliferator-Activated Receptor Gamma	PPAR-γ	100.92
2	Sterol Regulatory Element-Binding Proteins	SREBP	104.75
3	Nuclear Factor-κβ	NF-κβ	108.43
	**Inflammatory Markers**		
1	Tumor Necrosis Factors-α	TNF-α	98.71
2	Interluekin 6	IL6	96.84

Table S3. Experimental design employed in the study.

Days	HC	NIC-STZ	MET	SWE	SHAE	SAE
1st day	I. p. injection of saline solution and citrate buffer	I. p injection of nicotinamide (110 mg/kg) and STZ (65 mg/kg)	I. p injection of nicotinamide (110 mg/kg) and STZ (65 mg/kg)	I. p injection of nicotinamide (110 mg/kg) and STZ (65 mg/kg)	I. p injection of nicotinamide (110 mg/kg) and STZ (65 mg/kg)	I. p injection of nicotinamide (110 mg/kg) and STZ (65 mg/kg)
22nd day	–	Confirmation of stable hyperglycemia	Confirmation of stable hyperglycemia	Confirmation of stable hyperglycemia	Confirmation of stable hyperglycemia	Confirmation of stable hyperglycemia
23rd day	–	–	Metformin (200 mg/kg body weight, Per os)	Strawberry water extract (2 g/kg body weight, Per os)	Strawberry hydro-alcoholic extract (2 g/kg body weight, Per os)	Strawberry alcohol extract (2 g/kg body weight, Per os)
30th, 36th, 43rd day	Intermittent blood collection					
50th day	Blood samples were collected, animals were sacrificed. Liver, pancreas, brain, and kidney were harvested					

HC: Healthy control, NIC-STZ: NIC-STZ treated control, MET: Metformin-treated control, SWE: Strawberry water extract, SHAE: Strawberry hydro-alcoholic extract, SAE: Strawberry alcohol extract.

Table S4. Primer sequences of selected RT-PCR genes.

Sr. No.	Target Genes	Primers	Sequence
	**Housekeeping Gene**		
1	Glyceraldehyde-3-phosphate dehydrogenase	Forward	AGTTCAACGGCACAGTCAAG
		Reverse	TACTCAGCACCAGCATCACC
	**Fatty Acid Metabolism Genes**		
1	Carnitine Palmitoyltransferase 1A	Forward	CACTGATGAAGGAAGAAGAC
		Reverse	CCAGTCACTCACGTAATTTG
2	Malonyl CoA: ACP Acyltransferase	Forward	AAAACTCTAGGCTCAATCAAC
		Reverse	GGATGTGTGTATTTATGCCC
3	Acetyl-CoA Carboxylase Alpha	Forward	AGCAGTATTTGAACACATGG
		Reverse	CAGTTCCAAGAAGTAGAAGC
4	acyl-CoA Synthetase Long-chain family member 1	Forward	ACATTATGAACGATTGCTCC
		Reverse	GCATTACACACTCTACAACG
5	Fatty Acid Synthase	Forward	AAAAGGAAAGTAGAGTGTGC
		Reverse	GACACATTCTGTTCACTACAG
6	Fatty Acid Binding Protein	Forward	TGGAGGGTGACAATAAAATG
		Reverse	TCATGGTATTGGTGATTGTG
	**Transcription Factors**		
1	Peroxisome Proliferator-Activated Receptor Gamma	Forward	AAGACAACAGACAAATCACC
		Reverse	CAGGGATATTTTTGGCATACTC
2	Sterol Regulatory Element-Binding Proteins	Forward	AAACCTGAAGTGGTAGAAAC
		Reverse	TTATCCTCAAAGGCTGGG
3	Nuclear Factor-κβ	Forward	AAAAACGAGCCTAGAGATTG
		Reverse	ACATCCTCTTCCTTGTCTTC
	**Inflammatory Markers**		
1	Tumor Necrosis Factors-α	Forward	CTCACACTCAGATCATCTTC
		Reverse	GAGAACCTGGGAGTAGATAAG
2	Interleukin 6	Forward	CAGAGTCATTCAGAGCAATAC
		Reverse	CTTTCAAGATGAGTTGGATGG
